# Supplementary material for: Are carbon emissions trading and green financial instruments synergistic? -Comprehensive quantitative research based on content analysis
Source: PLoS One. 2024 Mar 7;19(3):e0298601. doi: 10.1371/journal.pone.0298601 (PMC10919601; doi:10.1371/journal.pone.0298601)
Supplement: S1 Data — (ZIP) [file pone.0298601.s001.zip › Data/地级市Z_F工作报告.zip!/░▓╗╒/╗┤▒▒╩╨/└┤╫╘╣1⁄2╓┌║┼í╛┬φ┐╦╩2╛▌═°í┐_2014.docx]

各位代表：
　　现在，我代表市人民政府，向大会报告工作，请予审议，并请市政协委员和其他列席人员提出意见。
　　2013年：攻坚克难、奋发进取
　　刚刚过去的2013年，面对宏观经济下行、煤炭价格大幅下降的复杂严峻形势，全市人民在中共淮北市委的坚强领导下，认真贯彻落实党的十八大和十八届三中全会精神，坚定信心，知难而进，围绕建设精致淮北谋篇布局，着眼长远做规划打基础，优环境抓项目，调结构促转型，惠民生筑和谐，经济社会实现持续健康平稳发展。预计全市地区生产总值650亿元，增长9 %；在煤炭税收同比下降27%的情况下，完成财政总收入93.03亿元，比上年下降8 %，其中地方财政收入50.68亿元，下降2.3%；规模以上工业增加值505亿元，增长10.1%，其中非煤产业增加值284亿元，占全部工业的56.2%，同比提高10.9个百分点；固定资产投资692亿元，增长20%；社会消费品零售总额195亿元，增长13.9%；外贸进出口总额4.4亿美元，增长24%；城镇居民人均可支配收入22500元，增长10.5%；农民人均纯收入8500元，增长13%。城镇新增就业4.5万人，城镇登记失业率4.1%；节能减排完成省下达的目标任务。
　　一年来，我们积极争取、主动跟进，一批事关全局的重大项目取得新突破，一批招商引资项目签约落地。安徽（淮北）新型煤化工合成材料基地产业发展规划获省政府批准，上升为全省煤化工发展重点。大唐虎山电厂投产发电，填补了我市作为能源基地无大型火电机组的空白，总投资58亿元的平山电厂获国家发改委核准正式开工建设。符夹线扩能暨高铁联络线项目得到国家铁路总公司认可，正待批复立项。我市跻身全国第二批智慧城市试点。再次荣获全国科技进步先进市称号。
　　一年来，我们主要做了以下工作：
  （一）坚持规划先行，引领城乡发展。为优化城乡空间布局，编制完成全域空间统筹规划，与皖北城镇群城镇体系规划对接。按照地上地下统筹、产城一体、组团式发展的思路，积极谋划城市发展的重点。完善了综合交通发展和城乡公共交通一体化发展规划。按照“调、输、蓄、用、节、净、美”统筹的思路，编制完成水资源利用与水环境治理规划。淮水北调淮北市配水工程方案、南湖和东湖景区规划通过专家评审，旅游、城市商业网点规划即将编制完成。出台城市供水、燃气、排水防涝、道路绿化、电网配套和棚户区改造等专项规划。调整了市开发区新区总体规划和龙湖片区控制性规划，完善了临涣煤化工产业园与浍河新城联动发展规划。完成一县三区村庄布点规划和37个中心村建设规划。按照市县一体原则，开展濉溪县城总体规划修编工作。这些规划相互衔接，合理布局生产、生活和生态空间，符合中央和我省关于城镇化工作的总体要求。
   （二）狠抓项目推进，扩大有效投入。坚持把项目建设作为稳增长、调结构、促转型的重要抓手，完善市领导联系督查重大项目机制，全年组织实施重点项目391个，完成投资417.3亿元。围绕完善城市功能，着力推进重大基础设施项目建设。省道235市开发区新区至泗许高速连接线、省道101平山口至徐楼段改建工程全面开工，省道202百善至涡阳段复工建设，南坪港一期工程主体完工。淮海东路等10条道路建成通车，沱河路等16条道路绿化工程全面完成，铺设供水管网67公里、污水管网50公里、天然气管网21公里。首座220千伏文昌宫智能变电站投运。总投资3.9亿元的火车站及广场建设基本完工，即将投入使用。淮矿集团工程建设公司科技大厦1号楼工程荣获中国建筑工程“鲁班奖”。围绕发展优势传统产业和新兴产业，积极推进重大产业项目建设。华中天力铝业、相邦复合材料、圣祥食品等项目开工建设，阳光机电、海聚科技园等项目加快推进，美信铝业、金龙机电等项目竣工投产。战略性新兴产业产值超过300亿元，增长25%，增速居全省前列。全年新增国家级高新技术企业23家。围绕推动服务业提速增效，规划建设一批现代物流、信息服务、文化旅游等服务业项目。百善物流园获批省级服务业集聚区，淮矿物流园一期基本建成，大润发二店、红星凯越广场开业运营，政通梦幻王国旅游项目开工建设，国购汽车文化产业园、口子文化博览园、四季榴园建设进展顺利。华东区互联网数据中心项目落户市开发区新区。新增限额以上流通企业108家，总数达339家。服务业完成增加值175亿元，增长8%。围绕提升农业发展水平，加快现代农业项目建设。完成农田水利基本建设投资2.1亿元，新增和改善灌溉面积29万亩、除涝面积45万亩，建设高标准农田2.9万亩。新增高效规模化种养基地110个，全市粮食总产120.1万吨，实现“十连丰”。规模以上农产品加工企业累计达到197家，实现产值428亿元，增长26%，增幅居全省前列。围绕改善生态环境，大力实施环境综合整治项目。投入资金1.37亿元，完成东湖、南湖区域地质环境治理和龙岱河等3条河道治理工程。加快推进37个中心村规划建设，完成投资8.7亿元。榴园、南山、郭王、黄新庄等一批特色精品村庄成为全省美好乡村建设的亮点。全面启动创建全国绿化模范城市工作。超额完成省下达的森林增长工程年度任务。
   （三）加大改革力度，创新体制机制。出台加快政府性投资项目建设意见，完善工程建设招投标管理办法，改变“八分开”缺少统筹的状况，逐步提高城市建设效能。调整征收安置补偿办法，健全拆违控违机制，积极化解城市建设中遇到的矛盾和问题。完善市土地管理委员会工作机制，开展土地清理专项行动，进一步规范土地利用。深入推进基层医药卫生体制改革，县级公立医院全部实行药品零差率销售。“营改增”试点稳步开展。完善财政资金管理使用办法，盘活存量资金，提高使用效益。推进政府投融资体制改革，成立市融资委，规范政府融资行为。组建交通、水务、南湖和临涣工业园投资公司，积极拓展融资渠道。持续推进金融创新。交通银行、招商银行在淮设立分支机构，同创担保公司完成增资扩股，濉溪县农村信用社改制工作稳步推进，淮矿集团财务公司、湖商村镇银行获准筹建，龙盘工贸等3家企业进入资本市场。制定林权抵押、专利权质押贷款办法，支持企业以股权质押、商标专用权质押、动产抵押等多种方式融资。完善考核奖励办法，引导金融机构扩大信贷投放，全年新增贷款103亿元，增长20%。大力推进产学研协同创新，与上海交大、中国林科院建立全面合作关系。创新农业生产经营体制，大力培育新型经营主体，新增流转土地10.8 万亩，专业合作组织发展到1100家，家庭农场达到269家。相山区获批全省服务业综合改革试点。烈山区成为全省农村综合改革示范试点区。
   （四）多措并举帮扶企业，壮大提升实体经济。加强与在淮央企和省属大企业战略合作，及时帮助解决企业遇到的困难。加快推进“百亿口子”行动计划，扶优扶强重点骨干企业。出台促进经济持续健康较快发展的46条意见和大力发展民营经济的82条措施，全力以赴保企业、稳增长。全面落实“五缓两降两补贴”政策，困难企业缓缴社保费3.68亿元，累计减收社保费6330万元，为57家企业发放就业岗位补贴1.25亿元。扎实开展“干部帮联企业，打造精良产业”行动，帮助中小企业解决融资、用地、用工等方面难题185个，落实贷款11.6亿元。启动跨区域进高校引智工程，为企业引进70多名高端人才。积极推进工业强市战略，深入实施“万千百十”工程，新增规模以上工业企业95家，总数达714家；新增产值超亿元企业72家，总数达392家。落实省市专项扶持资金1.44亿元，用于扩大融资担保金，支持小微企业和民营经济发展。大力实施创业富民工程，开展以创业带动就业富民行动，完成创业培训7900人，新增私营企业2102家、个体工商户12587户。民营经济占地区生产总值的比重达43%，同比提高1.5个百分点。
   （五）不断优化投资环境，持续推进招商引资。大力精简行政审批事项，市级行政审批项目从261项减少到157项，精简率达39.8%。优化再造审批流程，全市43家行政审批单位申报材料精简率、办理环节精简率、承诺时限压缩率分别为12%、19%和31%。免收市、区开发园区企业39项行政事业性收费和政府性基金，减轻企业负担2900万元。对招商引资项目实行招商单位全程帮办制度，推进“一会制”、“一表制”并联审批。加快园区载体建设，夯实招商平台，承接产业转移的能力不断增强。市开发区新区成为全省产城一体化试点园区。龙湖省级高新区获准筹建。濉溪开发区成为全省创新型园区，获批省级铝基新材料高新技术产业基地。凤凰山开发区获准扩区并成为省级科技园区。杜集开发区成为全省新型工业化产业示范基地。濉芜产业园建设与招商同步推进。调整完善招商引资优惠政策和考核办法，瞄准北京、上海、江苏昆山、浙江、广东和台湾等地，突出领导干部带头招商、以商招商、产业链招商，依托帮联企业招商。全市引进投资规模亿元以上新建项目102个，合同引资额405亿元。其中引进工业项目67个，商贸物流项目22个，其他服务业项目13个。与中建七局、中水电十三局、中铁四局等央企及北控水务合作共建基础设施项目成功签约，近期将陆续开工。全市累计引进市外资金330亿元，增长43.5%；实际利用外资4.5亿美元，增长 20%。坚持走出去、请进来，积极参与中原经济区、淮海经济区协作，扩大了城市开放度，提升了城市知名度，获得了大量的产业发展和项目信息，形成与央企、知名民企和外资、台资合作发展新机制，搭建了区域交流合作新平台。
   （六）大力改善民计民生，提升社会治理水平。在财政收入大幅减少的情况下，坚持民生优先，压减一般性支出。全市财政用于民生领域的支出 81亿元，占财政总支出的80%。34项民生工程全面完成。落实义务教育保障经费1.87亿元，惠及学生23万余名。发放各类教育资助资金6446万元，资助学生8.8万人次。18个乡镇公办中心幼儿园全部建成使用。72.7万人参加城乡居民养老保险，52.5万人参加城镇居民医疗保险，实现了全覆盖。城镇基本医保实现省内异地结算。新农合参合率全省第一，率先实现市级统筹。全年发放各类社会救助金2.97亿元，救助困难群众125.5万人次。新增养老机构床位3075张。新建30个村级组织服务用房。94个广播电视“村村通”工程、6个公共文化服务信息化建设项目全部完工，34个公共文化场馆和516个农家书屋全部免费开放。提前两年完成省定农村清洁工程建设任务。农村饮水安全工程全面完成，受益群众11.7万人。改造完成农村公路危桥18座。把解决22663户被拆迁户安置问题作为最紧迫的民生工程，采取有力措施集中解决难题、扫除障碍、理顺机制，加快24个安置点293万平方米安置房建设。新建保障性住房和棚户区改造房19047套，基本建成12421套。充分发挥住房公积金制度的保障功能，帮助9452个家庭改善了住房条件。社会事业协调发展。合理配置主城区教育资源，重新划分义务教育学区，实行中小学校阳光招生，有效解决了大班额现象和择校问题，体现了教育公平。高质量完成学前教育三年行动计划各项任务。市职教园区建设进展顺利，累计完成投资5.7亿元。农民文化乐园建设试点稳步推进。市图书馆获评国家一级图书馆。市妇幼保健院二期工程开工建设，市中医院跻身国家三级甲等中医医院。全市乡镇卫生院、村卫生室全部达到规范化建设标准。扎实推进大运河保护和申遗工作。我市健儿在全国第十二届运动会上摘得2金1铜，省羽毛球队落户淮北。积极开展城乡结合部环境综合整治，扎实推进文明创建活动，城市形象进一步提升。社会治理不断创新。出台政府购买社会组织服务办法，努力为群众提供更多更好的公共服务。加快农村社区建设，村级组织公共管理和服务水平全面提升。杜集区成为全国农村社区建设实验全覆盖示范单位。开展用群众工作统揽基层信访工作试点，扎实推进“领导干部大接访”活动，妥善处理群众信访矛盾纠纷，积极化解信访积案。社会治安防控体系不断强化，“天网”一期工程全面建成。以实行居住证制度为主要内容的户籍制度改革走在全省前列。深入开展安全生产大检查，推进“打非治违”和隐患排查，安全生产形势总体稳定。对重大食品药品安全事故实行“一票否决”，保障人民群众饮食用药安全。全面完成省政府下达的各项人口计生工作指标。国防教育、双拥和人防工作得到新加强。民族宗教、外事侨务、机关事务管理、保密、档案、地方志等工作取得新成绩。妇女、儿童、青少年、老龄、残疾人和慈善等事业实现新发展。
　　各位代表！过去的一年，我们坚持强化政府自身建设，认真落实中央和省、市委关于改进工作作风、密切联系群众的各项规定，着力破解制约科学发展的突出问题，认真解决群众反映的热点难点问题。建立邀请市人大、市政协领导列席市政府常务会议制度，自觉接受人大法律监督、政协民主监督和社会舆论监督。办理市人大代表议案、建议110件和政协委员提案186件，满意度99%以上。加大政务公开力度，推进网络问政，办好政风行风热线和说办就办栏目。办理“市长信箱”和网上留言938件，回复率97.5%，荣获人民网网民留言办理工作先进市称号。完善重大行政决策机制，规范政府决策行为，让权力在阳光下运行。强化目标管理，集中开展“大督查”活动，促进重大决策落实。继续对全市机关、公共服务单位及128名科（处）长开展民主考评，强化问责问效。加强对重点工程、关键领域的行政监察和审计监督，从严查处违纪违法案件。认真履行“一岗双责”，进一步深化廉政风险防控工作。严格落实国务院“约法三章”， “三公”经费等一般性支出同比下降14.2 %。全面开展行政事业单位国有资产清查工作，为加强管理、推进改革奠定了基础。  
　　各位代表！过去的一年，面对复杂严峻的经济形势，我们遵循经济发展规律，保持定力，沉着应对，推动淮北开放转型向前迈出了重要一步。这一年，市委坚强领导、统揽全局、科学决策，提出了建设精致淮北的奋斗目标，更加明确了淮北的发展方向；这一年，市人大、市政协对政府工作理解支持、有效监督，为淮北的发展集聚了正能量；这一年，全市广大干部群众齐心协力、攻坚克难、奋发进取，为淮北的发展作出了新贡献！在此，我代表市人民政府，向全市广大工人、农民、知识分子、干部和离退休老同志，向各民主党派、工商联、无党派人士、各人民团体，向驻淮人民解放军、武警官兵和公安干警，向在我市创业的投资者、建设者，向关心、理解、支持淮北发展的社会各界人士，表示衷心的感谢！
　　回顾一年来的工作，我们也清醒地认识到，我市经济社会发展中还存在许多困难和问题，主要表现在：产业结构性矛盾十分突出，财政收入出现负增长，部分企业生产经营困难，新的财税增长点还不多，服务业发展相对滞后、比重偏低，老城区拥挤需要疏解，新城区组团需要配套，政府职能转变还不到位，少数公务人员的进取和服务意识不强，解决问题的能力和办事效率不能适应我市转型发展的需要。我们将高度重视这些困难和不足，采取切实有力措施推进解决。
2014年：改革创新、稳中求进
　　2014年是贯彻落实党的十八届三中全会精神、全面深化改革的第一年，也是为打造精致淮北、全面建成小康社会奠定坚实基础的重要一年。纵观形势，我们面临的困难和挑战仍然较多。世界经济低速增长的态势仍在延续，我国经济进入深度转型调整阶段，我市产业结构调整、转型升级的任务比任何时候都更为迫切、更加艰巨繁重。但是，调结构需要战略定力，转方式需要历史耐心，急不来，也慢不得。面临新形势，我们的发展思路、工作套路和心理预期都要作出相应调整，需要更加自觉、更加主动地加快调结构转方式，推动经济提质增效升级，而不是片面追求增速拼资源，甚至牺牲环境，得不偿失。思路调整不过来，就很难适应新形势，新变化。同时，我们应该看到，党的十八届三中全会对全面深化改革作出总体部署，必将进一步增强经济社会发展的动力和活力，为我市破解发展难题创造良好的宏观环境。我们还要看到，我市整体纳入中原经济区规划，国家加大对资源衰退型城市可持续发展的支持力度。我们还拥有老工业基地调整改造、促进皖北振兴、全省新型城镇化试点等多项政策机遇。经过50多年的建设，我市产业发展基础、城市文明程度和人文环境等优势比较明显。市委、市政府提出建设产业精良、文化精深、城市精美、作风精细的精致淮北，到2020年与全国同步建成小康社会的奋斗目标，进一步明确了提升经济质量、加快转型发展的总体思路。这些目标和思路以及据此形成的一系列规划，已成为全市上下的共识，正在转化为推动淮北加快转型发展的强大动力。特别是经过去年招商引资和推进项目的前期工作，今年将有大批项目开工建设，推动项目引进转化为项目建设收益，提升经济发展的内驱力和支撑力。只要我们切实增强紧迫感和责任感，始终保持开拓进取的昂扬锐气，坚定信心，抢抓机遇，攻坚克难，就一定能够夺取新一轮发展的主动权。
　　今年政府工作的总体要求是：认真贯彻落实党的十八大、十八届三中全会以及中央、全省经济工作暨城镇化工作会议精神，按照市委七届七次全会的总体部署，坚持稳中求进的工作总基调，把改革创新贯穿于经济社会发展各个领域各个环节，牢固树立大开放、市场化、规划引领和可持续发展理念，以推进开放转型发展为主线，以提高经济增长质量和效益为中心，以招商引资和项目建设为抓手，以调结构转方式为主攻方向，更加注重改革创新增活力，更加注重改善民生促和谐，努力推动全市经济社会持续健康平稳发展，在打造精致淮北、全面建成小康社会征程上迈出新步伐。
　　今年经济社会发展的主要预期目标是：地区生产总值增长10%以上，财政收入增长3.2%，规模以上工业增加值增长12%，固定资产投资增长20%，社会消费品零售总额增长12.5%，外贸进出口总额增长15%，城镇居民人均可支配收入增长11%，农民人均纯收入增长12%，城镇新增就业3.5万人，城镇登记失业率控制在4.1%以内，人口自然增长率控制在9‰以内，节能减排完成年度目标任务。
　　重点做好六个方面工作：
　 （一）围绕释放发展活力，着力推进改革创新。深化行政体制改革。加快转变政府职能，正确处理政府与市场、与社会的关系，深入推进政企、政资、政事、政社分开，把政府工作重点转移到创造良好发展环境、提供优质公共服务、维护社会公平正义上来。以市、县政府机构改革为契机，理顺部门职责关系，优化工作流程。严格控制机构编制，确保财政供养人员总量只减不增。进一步简政放权，深化行政审批制度改革，减少和规范行政审批事项，简化办事程序，压缩审批时间，提高办事效率。实施工商、质监行政管理体制调整和食品药品监管体制改革。加快推进事业单位分类改革，培育发展各类中介组织。推进财政和投融资体制改革。实施全面规范、公开透明的预算制度，在全市首批44家预算单位开展预决算和“三公”经费信息公开。将财政资金扶持企业发展改变为贷款贴息等方式，并与企业纳税挂钩，主动接受社会监督。继续做好“营改增”扩围试点。稳步推进市、区财政体制改革，科学界定市、区事权、财权，促进财政支出责任明确、收益共享、协调增长。积极引进金融机构来淮设立分支机构，加快发展地方金融体系，确保濉溪农商行年内挂牌，湖商村镇银行开业运营。加强政银企对接，继续推广中小企业助保金、农村土地承包经营权、林权、专利权抵质押贷款，力争全年新增贷款110亿元。积极做好企业上市和中小企业集合票据发行工作，扩大直接融资规模。强化政府投融资平台建设，着力提高投融资运作水平。加强政府性债务管理，完善“借用还”一体化良性循环机制。加快推进其他领域改革。巩固和完善县级公立医院综合改革。理顺城市建设管理体制机制，规范政府性投资项目管理，创新征收工作方式。有序推进政府购买公共服务。加快环卫作业市场化改革。完善国有资产监管体制和经营机制，推进国有资产资本化。严守底线，严控总量，盘活存量，促进节约集约利用土地。全面激发市场主体活力。加快构建有利于全民创业的体制机制，毫不动摇鼓励、支持、引导民营经济发展。支持民营企业参与国企改革，积极发展混合所有制企业。推动民间资本投向基础设施、基础产业、金融服务、节能环保和教育、医疗卫生、健康养老等领域，进一步释放民间投资潜力。实施创业富民提升工程，完善和落实税收优惠、小额担保贷款、“个转企”等扶持创业政策，培育壮大各类市场主体，力争全年新增私营企业1600 户、个体工商户1.5万户，民营经济占地区生产总值比重达到44%。
   （二）围绕增强发展后劲，着力抓好项目建设和招商引资。加快推进项目建设。实施重点项目426个，年度计划投资498.1亿元。开工建设符夹线扩能暨高铁联络线、济祁高速淮北段、市区至临涣煤化工合成材料基地快速通道等重大交通项目和南坪船闸工程、淮水北调淮北市配水工程，确保省道235市开发区新区至泗许高速连接线、省道101平山口至徐楼段改建工程以及省道202线百善至临涣段、纵楼至孙谢庄段续建工程和平山口至西山隧道改建工程主体完工。加快建设平山电厂一期、淮海实业煤矸石建材综合利用和口子工业园东山项目，积极做好淮宿机场、平山电厂二期、国安电厂二期、中利电厂三期、任楼煤矿改扩建、国电风力发电等重大项目的前期工作，确保华孚工业园全面投产。推动实施理士电池等200个重点技改项目，提升企业核心竞争力。加大重点项目督查调度，有效破解用地、资金、环境等瓶颈制约，确保项目顺利实施。全力推进招商引资。围绕食品和新型煤化工、电子信息、高端装备制造、新材料、节能环保、生物医药等重点产业，抓好延链补链、产业集群和配套服务招商。紧盯世界500强、国内100强和行业10强企业，及时捕捉投资动态，主动出击，着力引进大企业、大项目。以完善城市功能为重点，加快引进一批商贸物流、金融服务、文化旅游、健康养老等生产、生活性服务项目。聚焦重点区域科学招商，把北京、上海、浙江、广东、江苏昆山、台湾等地作为引进央企、外企、民企和台企的重点，锲而不舍，持续跟进。在开展产业链招商、以商招商的同时，强化专业招商，积极开展委托招商和园区共建招商。全面落实39项行政审批费用减免政策，加快准入项目审批，完善开发园区“封闭运行”机制，加快建设标准化厂房、现代物流等配套设施，为招商企业落地创造良好环境。加强项目预审，把好节约集约用地、环境保护和对财税的贡献关。完善招商项目调度机制，推进在谈项目尽快签约，签约项目尽早落地，力争全年引进固定资产投资亿元以上项目100个。大力提升园区建设水平。加快市开发区“二次创业、转型升级”，促进产业集聚、资源集约、产城融合。优化市开发区新区工业、商贸、公共服务空间布局，完善交通、供电、通信等配套设施，着力打造产城一体、四化同步、宜业宜居的工业新城。多渠道筹措建设资金，加快临涣煤化工合成材料基地建设，完善道路、供水、供电等基础设施。争取落实直供电和自备电政策，提高招商引资的竞争力。完成濉芜产业园“十路四桥”等配套工程。提升濉溪开发区产业竞争力，积极争创国家级开发区。支持凤凰山经济开发区建成全国知名的专业园区。支持杜集经济开发区争创国家级高新技术产业基地。
    （三）围绕培育精良产业，着力推进产业转型升级。以扶持企业做大做强助推经济增长。深化与在淮央企、省属大企业对接合作，充分发挥大企业对全市经济发展的支撑和带动作用。积极推进“百亿口子”行动计划，支持口子酒业加快发展。以未来产出可达50亿元、100亿元以上的骨干企业为重点，集中力量打造一批领军企业。继续实施“万千百十”工程，支持中小企业发展，夯实精良产业支撑主体，力争全年新增规模以上工业企业100家、产值超亿元企业80家。继续深入开展“干部帮联企业，打造精良产业”行动，全力帮助企业解决发展难题。坚持引进来、走出去相结合，鼓励更多企业开拓国际市场，千方百计扩大外贸进出口总量。以加快发展优势产业引领转型升级。紧抓省委、省政府支持淮北发展新型煤化工的战略机遇，开工建设总投资147亿元的年产220万吨焦炭的煤焦化二期、年产10万吨的粗苯精制、年产30万吨的煤焦油及深加工、新型墙体材料等12个关联项目，全面拉开新型煤化工基地建设的序幕，拉长产业链，做大产业集群，力争到2020年，煤化工产业销售收入达到1000亿元，初步建成全国重要的煤化工产业基地和循环经济发展示范基地，成为推动淮北跨越崛起的引领工程。改造提升煤电传统产业，关闭淘汰落后的地方小煤矿。以龙头企业为支撑、重大项目为抓手，加快推进食品工业向精深加工发展、机械制造业向高端成套设备发展、纺织服装产业向高精细发展、新型建材产业向节能环保方向发展，提升产业发展层级和水平。培育壮大高端装备制造、生物医药、电子信息、新材料等战略性新兴产业，确保新兴产业产值增长20%以上。力争铝基新材料高新技术产业基地列入全省重点产业生产力布局和调整规划，争取更多的支持。以提高自主创新能力驱动结构调整。加快企业主体、创新载体和产学研一体建设，推动传统产业改造升级，力争全年新增高新技术企业15家，实现高新技术产业产值360亿元。突出人才与产业发展融合，指导市属高校调整学科设置，培养实用人才。强化人才第一资源理念，积极招才引智，依托高水平人才培育高新技术产业。加强与中科院、清华大学、上海交大、淮北师大等高校、科研院所合作，加快搭建新型煤化工、铝基新材料、电子信息等产业技术公共服务平台。启动国家知识产权示范市创建工作，力争全年发明专利申请量、授权量达到550件和85件。加快推进龙湖高新区和市科技企业孵化器建设。支持烈山区争创国家级可持续发展实验区。以大力发展现代服务业优化产业体系。继续实施服务业提速增效行动，力争服务业完成增加值190亿元，增长9%。加快老城区传统服务业提档升级，谋划建设龙湖高新区、职教园区和市开发区新区服务配套设施。继续推进新网工程和万村千乡市场工程，加快流通产业发展，不断优化消费环境。重点抓好淮北义乌小商品城、国购城市综合体和中泰广场等重大服务业项目建设。鼓励发展现代物流、电子商务等生产性服务业，加快推进青龙山商贸物流园、淮矿物流园二期、中易物流园建设。大力发展健康养老、科技研发、工业设计、休闲旅游等新兴服务业，强化旅游产业功能配套和景区景点建设，力促四季榴园成功创建国家4A级风景区。办好第三届食品工业博览会和第五届石榴文化旅游节。促进房地产业健康稳定发展。
    （四）围绕打造精美城市，着力提升城市建设管理水平。加快城市基础设施建设。加大老城区基础设施改造力度，有序引导公共资源向新城区转移。加快南湖景区建设，确保年内核心景区建成开放。强化城市路网建设与改造，逐步打通断头路，提高城区快速通达能力。加快实施东岗楼立交桥维修加固工程。持续开展城市道路绿化及供排水、燃气管网改造。指导各县区和在淮省属大企业有序推进棚户区改造。加强生态环境建设。以创建全国绿化模范城市为契机，抓好城区东部山场绿化和森林增长工程，确保完成1.6万亩荒山绿化和6.4万亩年度造林任务。推广芳香种植，为城市绿化增添色彩。加强矿山地质环境治理和生态修复。积极推进相阳沟等城市水环境综合整治。强化污水处理和中水利用，确保污水达标排放。加大清洁能源推广使用力度，抓好大气污染防治，有效改善空气质量。争创全国文明城市。着眼于提升市民文明素质，深入实施公民道德建设工程，放大道德模范和身边好人的榜样作用。进一步完善城市管理长效机制，实施城市管理示范街创建工程，积极开展市容市貌、交通秩序、环境卫生专项整治，着力提升城市管理水平，奋力争创第四批全国文明城市。坚持城乡同治、共建共享，动员全社会力量积极参与文明创建。加强社区环境综合整治，切实抓好基层创建工作。以服务市民、方便市民为目的，积极推进智慧城市建设，提升城市智能化、信息化水平。
　　（五）围绕强化农业农村发展基础，着力推动城乡发展一体化。积极推进新型城镇化建设。坚持以人的城镇化为核心，加快产城融合，统筹城乡发展，着力提高城镇化发展质量。进一步深化户籍制度和与之相挂钩的就业、社保等配套制度改革，有序推进农业转移人口市民化。加强农村建设用地整治，争取启动“人地挂钩”政策试点，增强用地保障能力。结合采煤塌陷区村庄搬迁安置和煤化工产业基地配套完善，谋划启动浍河新城建设，打造市域南部次中心。大力提升农业现代化水平。继续开展小麦、玉米高产创建活动，抓好“菜篮子工程”建设，提升养殖业规模档次，完善农业社会化服务体系，增强农业综合生产能力。加快构建南粮、北菜、东部特色水果的产业布局，着力发展高效、生态、特色农业。提升凤凰山等4个食品工业园区承载能力，加快发展农产品深加工业。重点培育发展100家农业龙头企业、100家农业合作示范社和50个市级示范性家庭农场，推进农业适度规模经营。加快百善等6个现代农业示范区建设，带动全市现代农业转型升级。扎实推进美好乡村建设。继续完善37个中心村基础设施和公共服务配套，确保8个省级重点示范村达标验收。规划建设一批具有地域特色、生态特色和人文特色的示范村，积极发挥示范村对全市美好乡村建设的带动作用。整合各类涉农项目资金，统筹使用专项资金支持美好乡村建设。突出“三线三边”，推进农村环境综合整治，实现中心村垃圾集中收集、污水有序排放。
　　（六）围绕保障和改善民生，着力加强和谐社会建设。巩固完善民生工程。按照保基本、补短板、兜底线、可持续的要求，既尽力而为又量力而行，务实安排民生工程项目，关注弱势群体，解决群众最关心的问题。对农村低保、贫困残疾人等涉及困难群体生活的项目扩面提标，稳步提高社会保障水平。大力培育精深文化。坚持用社会主义核心价值体系引领社会思潮、凝聚社会共识。实施文化强市战略，深化文化体制改革，促进文化事业全面繁荣。深入推进文化惠民工程，加大城乡公共文化设施建设力度，循序渐进建设农民文化乐园，促进基本公共文化服务标准化、均等化。积极做好大运河保护和申遗工作。大力扶持文化产业发展，重点抓好隋唐运河古镇文化旅游、濉溪文化产业园、国购汽车文化产业园、口子文化博览园等重大项目建设。实施文艺精品工程，扩大对外宣传和交流，不断提升淮北文化的知名度和影响力。统筹发展社会事业。多渠道开发就业岗位，提高城乡居民收入，重点解决好高校毕业生、农村转移劳动力、城镇困难人员、退役军人和残疾人就业。深化教育综合改革，促进各类教育全面协调发展。启动实施第二期学前教育三年行动计划，深入推进义务教育均衡发展。加强教师队伍建设，提升师德水平。规划建设市一中东校区，确保市职教园区一期工程9月1日前建成使用。巩固基本医保覆盖面，提高基本医疗保障和服务水平。推动市紧急救援中心和市人民医院东院区建设，支持市妇幼保健院、职防院争创三甲专科医院。支持驻淮部队建设，深化双拥优抚安置工作。积极备战全省第十三届运动会。加强人口和计划生育工作。认真做好第三次全国经济普查工作。充分发挥工会、共青团、妇联等人民团体的重要作用，推进科普、档案、地方志、儿童、老龄、慈善等事业发展，进一步加强民族宗教、外事侨务、气象、地震等工作。不断提升社会治理水平。改革信访工作制度，完善考评机制，加大领导干部接访下访力度，实行网上受理信访制度，全面推行用群众工作统揽基层信访工作，着力解决信访热点问题、遗留问题。深入开展“六五”普法活动，扎实推进“法治淮北”、“平安淮北”建设，健全社会治安防控体系，预防和依法惩治违法犯罪。加强农村社区建设，年内全面建成镇级社区综合服务中心。完善公共安全和应急管理机制，健全网络舆论引导机制、舆情监测体系。加强检验检测能力建设，强化食品药品安全监管。健全安全生产责任体系，进一步加强重点行业和领域安全隐患整治，加大“打非治违”力度，有效防范和遏制重特大安全事故发生。
　　各位代表！全面完成今年的各项目标任务，必须强化问题导向和责任意识，坚持政府工作项目化，项目工作责任化，进一步改进和创新政府工作，不断提升行政效能和服务水平。扎实开展党的群众路线教育实践活动，深入基层倾听意见，主动接受群众监督，着力解决与群众利益密切相关的热点、难点问题。大力弘扬真抓实干之风，发扬“钉钉子”的精神，敢于担当、勇于负责，切实把工作落到实处。精简会议文件，改进文风会风。严格政府绩效管理，加强机关效能建设，深入开展政风行风评议活动，加大治慵治懒治散力度，强化效能督查，通过干部作风转变促进政府职能转变。落实重大行政决策程序规定，完善行政决策风险评估机制，着力提高政府决策科学化、民主化、法制化水平。更加自觉地接受人大法律监督、工作监督和政协民主监督，认真办理人大代表议案、建议和政协委员提案，高度重视社会公众、新闻媒体和网络舆论监督。深入推进政务和办事公开制度，及时主动公开涉及群众利益的信息，向群众说真话、交实底。严格执行中央“八项规定”，厉行节约反对浪费。严控财政预算外支出，确保“三公”经费只减不增。全面落实党风廉政建设责任制，深入推进廉政风险防控，强化行政监察和审计监督，坚持用制度管人、管钱、管土地、管项目。坚决纠正损害群众利益的不正之风，严肃查办违纪违法案件，切实做到干部清正、政府清廉、政治清明。
　　各位代表！宏图凝聚人心，形势催人奋进。让我们在中共淮北市委的坚强领导下，凝心聚力，创新务实，锐意进取，为圆满完成本次大会确定的各项任务，为打造精致淮北、全面建成小康社会而努力奋斗!
